# Supplementary material for: Polyphenol-Rich Aronia melanocarpa Fruit Beneficially Impact Cholesterol, Glucose, and Serum and Gut Metabolites: A Randomized Clinical Trial
Source: Foods. 2024 Aug 30;13(17):2768. doi: 10.3390/foods13172768 (PMC11395532; doi:10.3390/foods13172768)
Supplement: Supplementary file 1 [file foods-13-02768-s001.zip › foods-3140473-supplementary.pdf]

Supplementary Material

Title: Polyphenol-rich *Aronia melanocarpa* fruit beneficially impact cholesterol, glucose, and serum and gut metabolites: a randomized clinical trial

Authors: Morgan Chamberlin[1†], Jesse T. Peach[2,3†], Stephanie M.G. Wilson[1,4,5], Zachary T. Miller[6], Brian Bothner[2], Seth T. Walk[7], Carl J. Yeoman[8], and Mary P. Miles[1*]

1. Supplementary Data

1.1 Metabolomic data: https://www.metabolomicsworkbench.org.

1.2 16S microbial taxonomy data: http://www.ncbi.nlm.nih.gov/bioproject/1136926, PRJNA1136926

2. Supplementary Figures and Tables

2. Supplementary Figures and Tables

Figure S1. CONSORT flowchart of study design and enrollment. Individuals were recruited using flyers and emails. Twenty-five individuals completed an initial eligibility screen by the research team. Fifteen individuals met study requirements and were randomly allocated to Aroniaor placebo groups and fourteen participants completed the study.

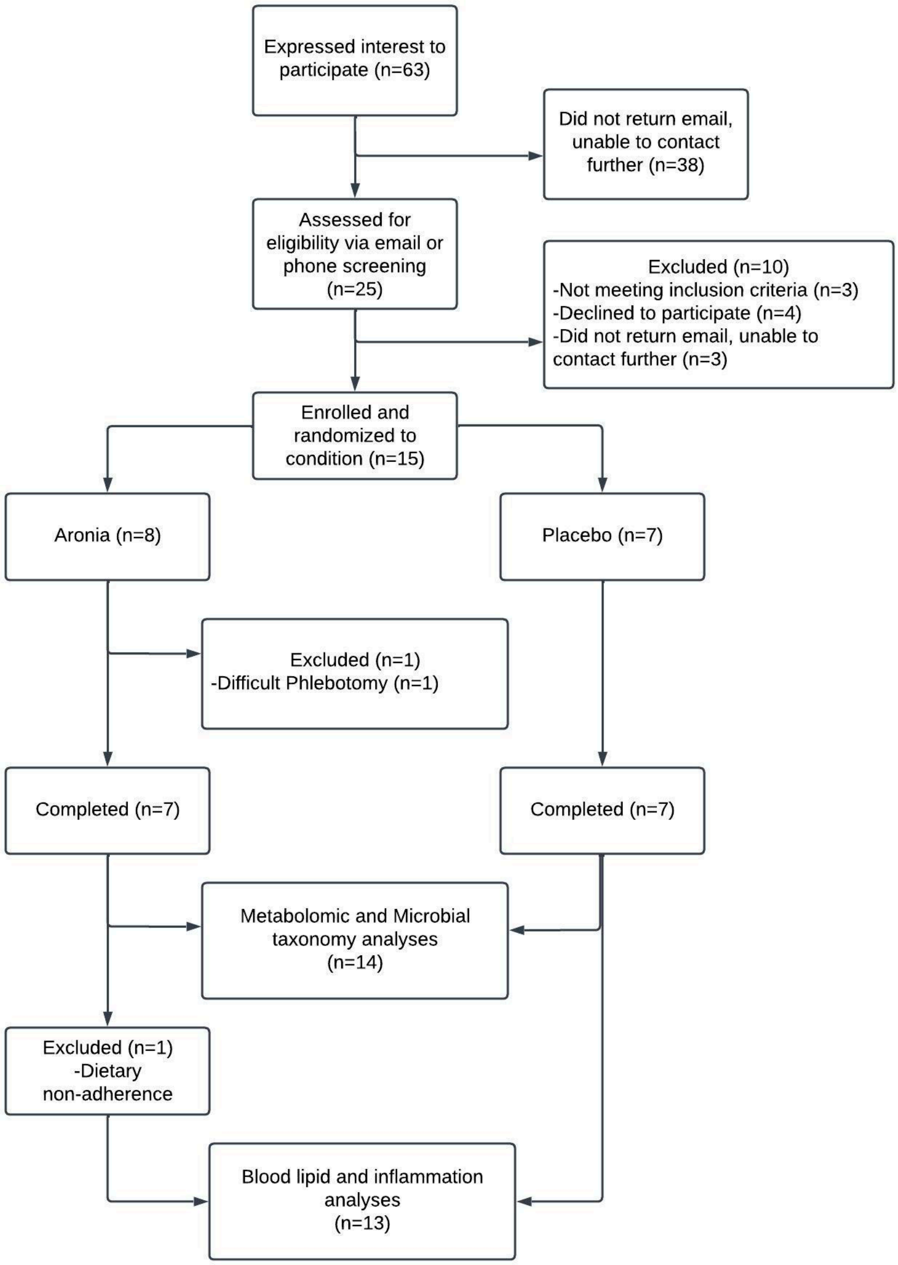

Supplementary Figure S2. Radar plot depicting habitual dietary intake of participants by intervention group at baseline as described by average HEI component scores. HEI component scores were calculated through the self-reported, online DHQ III dietary survey. Scores of adequacy include total fruit, whole fruit, total vegetables, greens and beans, whole grains, dairy, total protein foods, seafood and plant proteins and fatty acids. Scores of moderation include refined grains, sodium, added sugars and saturated fats. Points indicate group means. Total score possible for each category listed on chart.

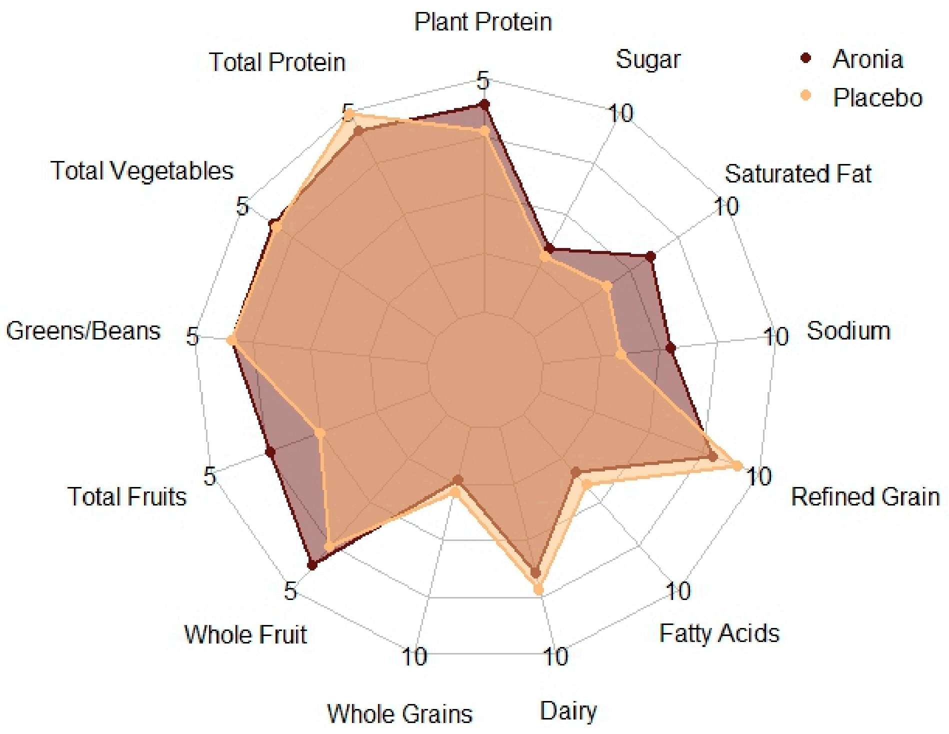

Supplementary Figure S3. Summary plot of individual changes (post-intervention–pre-intervention) in triglyceride area under the curve (dTG$_{AUC}$). dTG$_{AUC}$ values represent the change in the sum of values from fasting and hourly time points for 6 h post high-fat meal ingestion. Each bar is representative of the value for one participant (n = 13). Difference in dTG$_{AUC}$ between meal groups determined with ANOVA and displayed in inset.

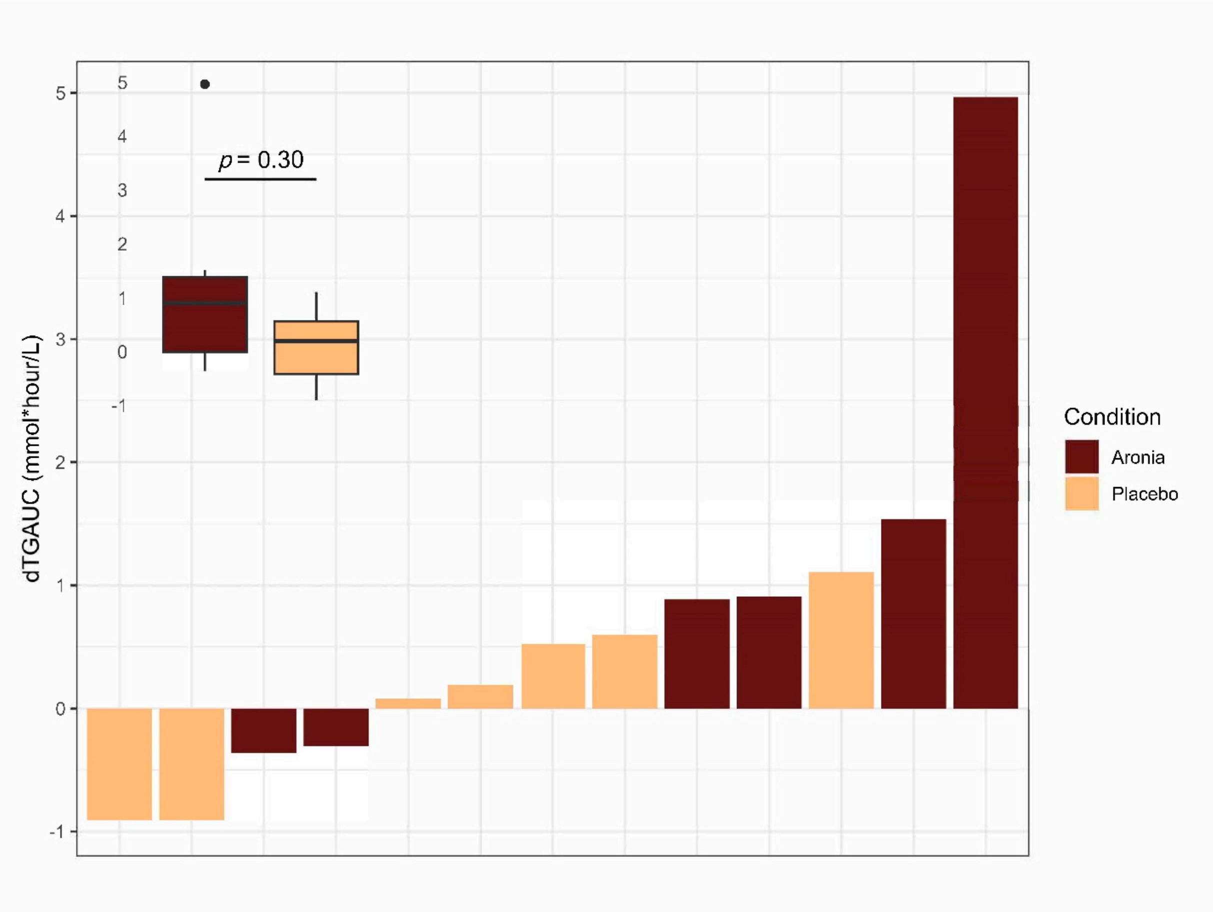

Supplementary Figure S4. Postprandial triglyceride (TG) response a high-fat meal (50 gram fat) before and after the 30-day intervention, by intervention group. Points indicate group means, bars represent ± standard error.

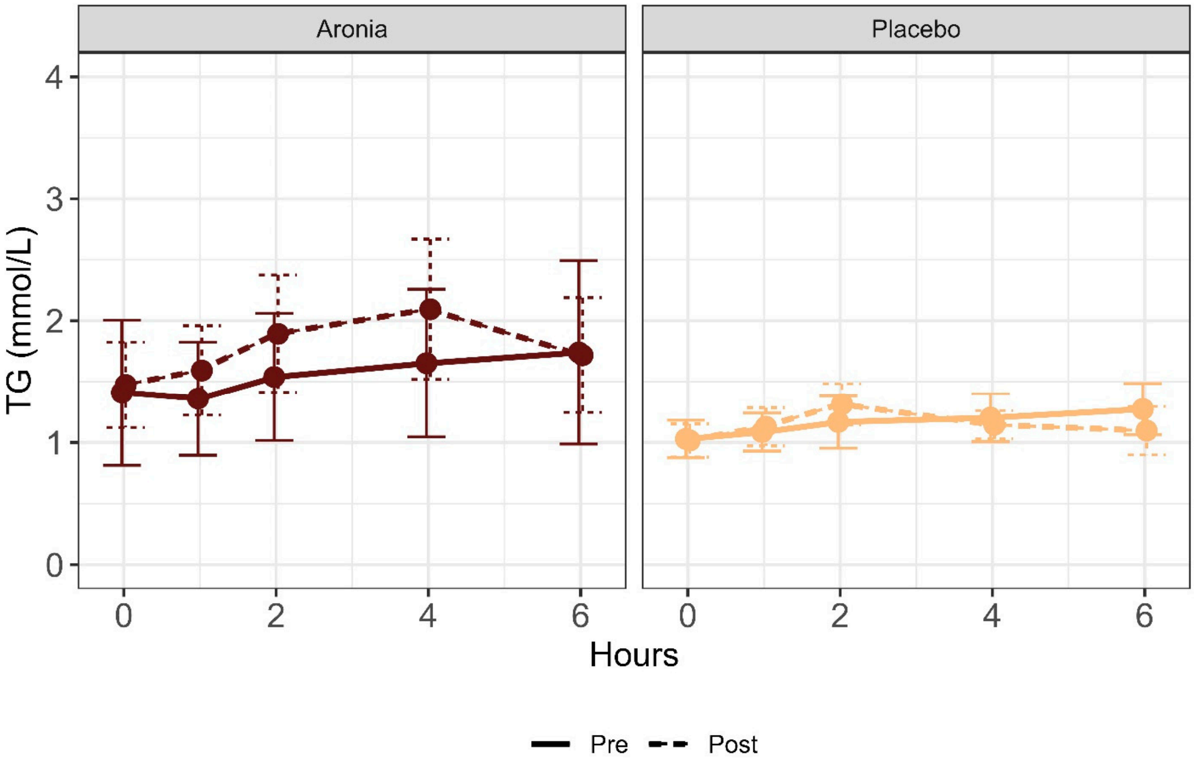

Supplementary Figure S5. Postprandial glucose (GLU) response to a high-fat meal (50 gram fat) before and after the 30-day intervention, by intervention group. Points indicate group means, bars represent ± standard error.

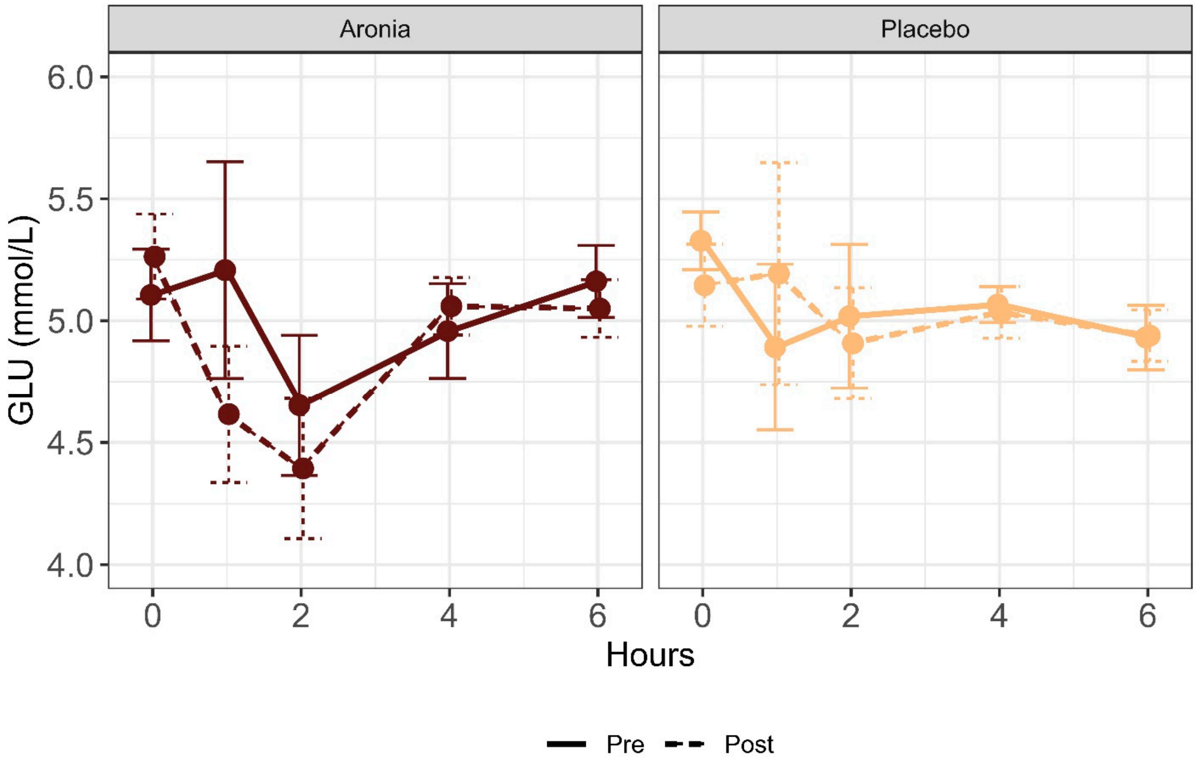

Supplementary Figure S6. PCA 2-D plot of global A) serum and B) fecal metabolites from pre- and post- intervention.

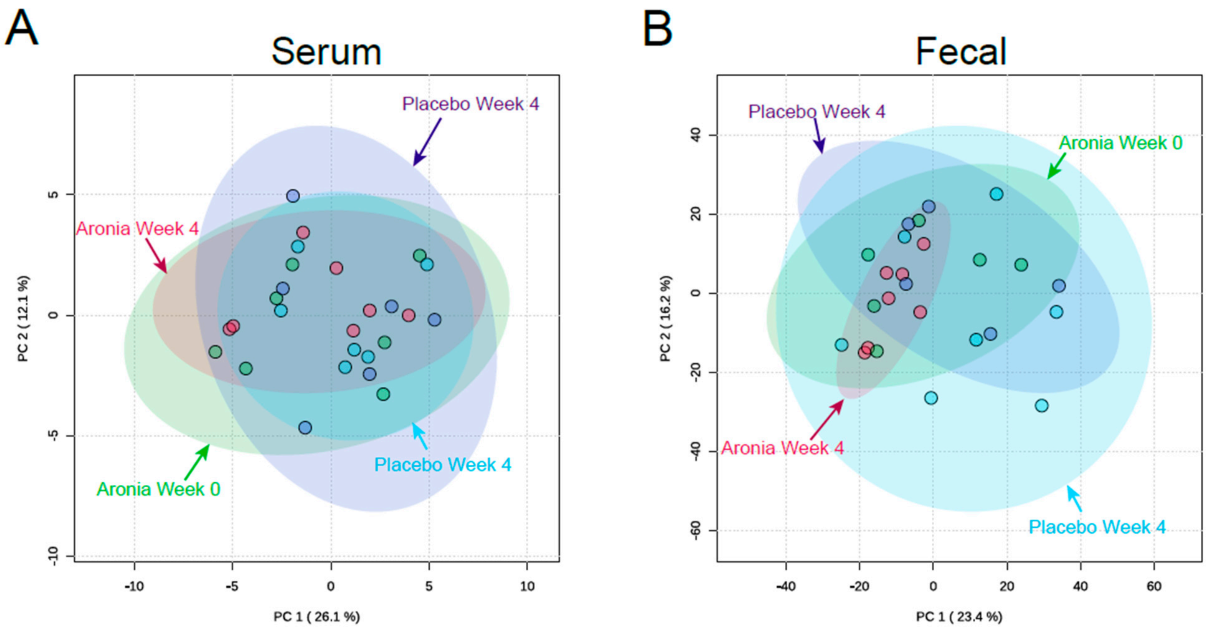

Supplementary Table S1: List of foods to avoid. List of foods provided to participants at first visit to avoid eating for one-week dietary washout period before second visit and for duration of the 30-day intervention period.

Berries	Fruit	Vegetables	Beans	Nuts	Other
Blueberries	Apples	Kale	Pinto	Walnut	Dark chocolate
Blackberries	Black Plum	Broccoli	Black	Pecan	Black rice
Strawberries	Prune	Beets	Red		
Cranberries		Artichoke	Kidney		
Cherries					
Raspberries					
Goji					

Supplementary Table S2. Baseline habitual dietary intake of intervention groups represented by Healthy Eating Index (HEI) total and component scores. HEI component scores were calculated through the DHQ III food survey during which participants self-report type and frequency of foods consumed. Bold p-values indicate strong evidence of a difference between meal groups.

**Component**	**Maximum points**	**Score**		
		**ARO (*n*=6)**	**PLA (*n*=7)**	***p*-value**
**Adequacy**				
Total fruits	5	3.7 ± 1.6	2.5 ± 1.4	0.20
Whole fruits	5	4.3 ± 1.3	3.8 ± 1.6	0.51
Total Vegetables	5	4.3 ± 1.4	4.2 ± 1.0	0.87
Greens and beans	5	4.2 ± 1.9	4.6 ± 0.9	0.52
Whole grains	10	2.4 ± 1.0	2.8 ± 1.6	0.52
Dairy	10	6.4 ± 1.1	7.2 ± 1.9	0.39
Total protein foods	5	4.5 ± 0.7	5.0 ± 0.1	0.15
Seafood and plant proteins	5	4.5 ± 1.1	3.9 ± 1.4	0.44
Fatty acids	10	3.3 ± 2.4	4.0 ± 3.4	0.68
**Moderation**				
Refined grains	10	7.9 ± 2.6	9.0 ± 1.3	0.35
Sodium	10	5.5 ± 3.6	3.4 ± 1.3	0.17
Added sugars	10	5.7 ± 3.4	4.5 ± 3.0	0.53
Saturated fats	10	6.1 ± 1.6	3.8 ± 1.7	**0.03**
**Total**	100	62.8 ± 7.0	58.7 ± 8.5	0.38

Data represent mean ± sd. Significance between group means determined with t-test.
Abbreviations: ARO, aronia; PLA, Placebo; DHQ, diet history questionnaire.

Supplementary Table S3. Change in fasting and postprandial inflammation by intervention group. Fasting values represent time point 0 collected prior to 50 gram, high-fat meal challenge. Postprandial values summarized as net area under the curve (AUC) using fasting and 1-, 2-, 4-, and 6- hour postprandial measurements. Delta values were calculated as (post-intervention – pre-intervention) and expressed by Δ Baseline to represent changes in fasting inflammation and Δ AUC to represent changes in postprandial inflammation.

	**ARO (*n*=6)**		**PLA (*n*=7)**		***p*-value**	
**Cytokine**	**Δ Baseline**	**Δ AUC**	**Δ Baseline**	**Δ AUC**	**Δ Baseline**	**Δ AUC**
IL-6	0.84 ± 1.15	11.49 ± 34.40	-0.14 ± 1.85	-3.42 ± 10.33	0.34	0.53
IL-1β	0.19 ± 0.49	-0.48 ± 3.15	-0.33 ± 0.60	1.50 ± 2.70	0.13	0.17
IL-17	1.13 ± 1.38	-1.22 ± 14.39	-0.17 ± 4.81	-16.56 ± 58.54	0.53	0.58
IL-23	27.20 ± 75.03	-114.03 ± 520.99	14.20 ± 88.89	73.04 ± 781.89	0.09	0.27
IL-10	2.98 ± 3.97	-13.94 ± 21.15	-3.40 ± 9.13	-10.83 ± 75.56	0.17	0.66
TNF-α	0.96 ± 1.38	-3.30 ± 8.73	-0.98 ± 3.01	6.95 ± 15.05	0.24	0.14
GM-CSF	1.84 ± 5.55	9.41 ± 41.23	-1.03 ± 20.44	-13.07 ± 98.92	0.78	0.68
IFN-γ	9.24 ± 21.76	-63.90 ± 136.44	4.56 ± 135.64	-542.55 ± 1678.36	0.90	0.43

Data represent mean ± standard error. Linear mixed modeling used to determine differences ($p < 0.05$) between group means.
Abbreviations: ARO, control; PLA, placebo; IL, interleukin; TNF-α, tumor necrosis factor alpha; GM-CSF, granulocyte macrophage colony stimulating factor, IFN-γ, interferon-gamma; AUC, area under the curve.

Supplementary Table S4. PERMANOVA results at each taxonomic level for the time and juice interaction. *P*-values and R^2 values reflect the interaction between juice condition and time.

Taxon	R^2	Pr (>F)	Features
Phylum	0.002	0.95	10
Class	0.004	0.97	22
Order	0.009	0.95	32
Family	0.004	0.99	55
Genus	0.009	0.99	160
HFE selected	0.011	0.947	89

Abbreviations: HFE, hierarchical feature engineering; OTU, operational taxonomic unit.

Supplementary Table S5. Microbial taxa associated with Aronia juice consumption before multiple testing correction. Taxa were identified using MaAsLin2.

Genus	Coefficient	Std. Error	*p*-value	q-value
Holdemania	-0.674	0.205	0.0064	0.82
Oxalobacteraceae unclassified	0.353	0.152	0.0387	0.82
Barnesiella	-0.312	0.141	0.0469	0.82
Prevotellaceae unclassified	0.546	0.263	0.0487	0.82
Pasteurellaceae unclassified	0.316	0.146	0.0508	0.82
